# Reativação da Sarcoidose Cardíaca em um Coração Transplantado: Um Caso Incomum de Nova Disfunção do Enxerto

**DOI:** 10.36660/abc.20250311

**Published:** 2025-11-27

**Authors:** Manuela Cristina Ribeiro Dias Barroso, Heleutério da Conceição Nicolau Madogolele, Danielle Louvet Guazzelli, Luis Fernando Bernal da Costa Seguro, Fabiana Goulart Marcondes-Braga, Sandrigo Mangini, Monica Samuel Ávila, Gabriel Barros Aulicino, Iascara Wozniak Campos, Samuel Padovani Steffen, Fabio Antonio Gaiotto, Fernando Bacal

**Affiliations:** 1 Hospital das Clínicas da Faculdade de Medicina da Universidade de São Paulo Instituto do Coração São Paulo SP Brasil Instituto do Coração do Hospital das Clínicas da Faculdade de Medicina da Universidade de São Paulo, São Paulo, SP – Brasil

**Keywords:** Sarcoidose, Transplante de Coração, Disfunção Primária do Enxerto

## Introdução

A sarcoidose cardíaca (SC) é uma manifestação incomum, porém potencialmente fatal, da sarcoidose sistêmica. A recorrência da SC após transplante cardíaco (TC) é um evento extremamente raro e representa um desafio diagnóstico significativo.

## Relato de Caso

Paciente do sexo feminino, 50 anos, admitida em nosso serviço por disfunção nova do enxerto, com redução da fração de ejeção do ventrículo esquerdo (FEVE) ao ecocardiograma transtorácico de rotina (de 64% para 45%), assintomática.

A SC foi diagnosticada aos 39 anos, se manifestou inicialmente com bloqueio atrioventricular de alto grau com implante de marcapasso aos 37 anos. O paciente desenvolveu insuficiência cardíaca avançada com fração de ejeção reduzida, com múltiplas hospitalizações, e permaneceu sintomática apesar do tratamento clínico otimizado. Aos 39 anos, foi realizado TC ortotópico bicaval.

O paciente apresentou uma recuperação precoce tranquila e sem intercorrências após o TC, com resultados consistentemente negativos na biópsia endomiocárdica (BEM) de vigilância e titulação gradual da ciclosporina. A terapia imunossupressora de longo prazo incluiu ciclosporina 50 mg. duas vezes ao dia, micofenolato de mofetil 500 mg duas vezes ao dia e prednisona 5 mg ao dia.

Dada a nova redução da FEVE em um coração transplantado, as hipóteses de rejeição do enxerto, infecção oculta, doença vascular do enxerto cardíaco (DEV) e reativação da SC foram consideradas.

Os exames de sangue iniciais revelaram proteína C-reativa <0,4mg/L [NR: 1-3mg/L], peptídeo natriurético tipo B de 380pg/mL [NR: <100pg/mL], troponina I ultrassensível de 17ng/L [NR <16ng/L] e nível sérico de ciclosporina de 210ng/mL. Eletrocardiograma em ritmo sinusal, prévio bloqueio de ramo direito e índice cardiotorácico normal na radiografia de tórax, com fios metálicos de esternotomia prévia.

**Figura 1:**
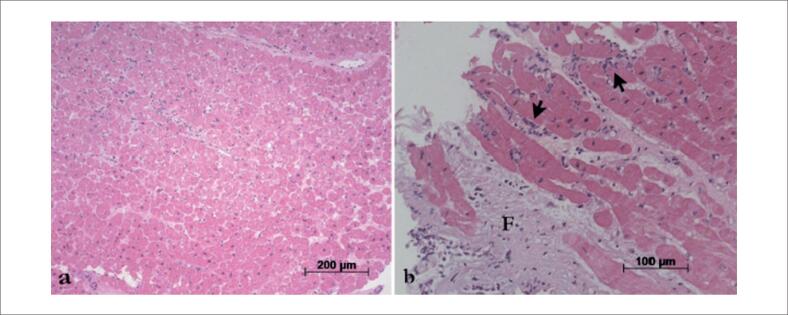
Fotomicrografias da última biópsia endomiocárdica mostrando (a) Ausência de infiltrado inflamatório ou fibrose. (b) Foco mínimo de lesão miocárdica (setas pretas) e fibrose leve (F). O grau de rejeição celular aguda foi de 1R. Os fragmentos foram seccionados em vários níveis, e não havia evidência de granulomas. Coloração com hematoxilina-eosina, aumentos das objetivas de 10X e 20X, respectivamente.

**Figura 2:**
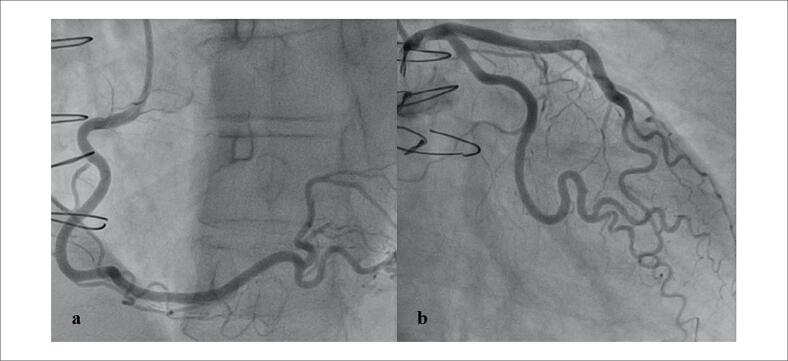
Angiografia coronária invasiva. (a) Vista oblíqua anterior direita mostrando a artéria coronária direita sem lesões obstrutivas significativas. (b) Vista caudal oblíqua anterior esquerda mostrando a artéria coronária esquerda, com as artérias descendente anterior esquerda e circunflexa livres de estenose crítica.

Investigações adicionais para um possível foco infeccioso oculto, incluindo exames de imagem, hemoculturas e uroculturas, apresentaram resultados negativos. Uma BEM foi então realizada, revelando rejeição celular 1R e nenhuma evidência de rejeição humoral (Figura 1), e o painel de anticorpos reativos não mostrou anticorpos específicos do doador. No cateterismo cardíaco, não havia evidência de DEV (Figura 2).

**Figura 3:**
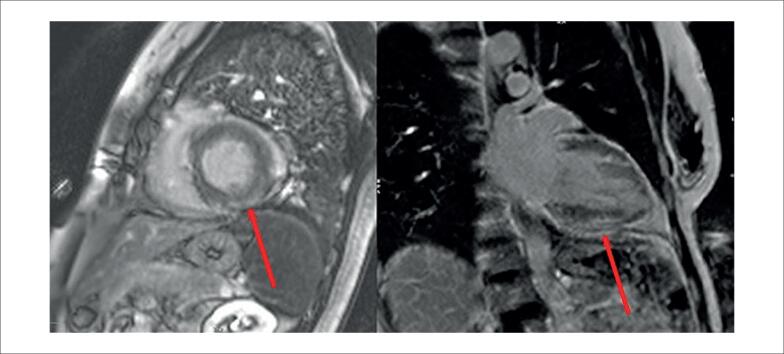
Ressonância magnética cardíaca mostrando edema e realce tardio multifocal de padrão meso subepicárdico não isquêmico, com predominância no ventrículo esquerdo inferosseptal, inferior e médio-basal inferolateral, compatível com miocardiopatia inflamatória (setas vermelhas).

**Figura 4:**
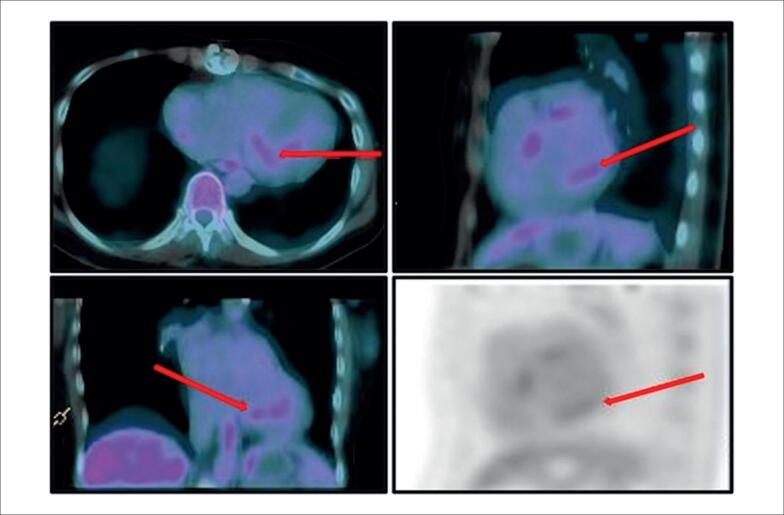
Tomografia por emissão de pósitrons/tomografia computadorizada com ^18^F-fluorodesoxiglicose demonstrando (setas vermelhas) captação discreta, heterogênea e multifocal do radiotraçador nos segmentos inferosseptal basal, inferior e inferolateral do miocárdio ventricular esquerdo (máximo valor de captação padronizado: 2,4), achados sugestivos de processo inflamatório ativo em andamento. As imagens foram adquiridas por meio de tomografia por emissão de pósitrons acoplada a tomografia computadorizada (tomografia computadorizada de 64 cortes), uma hora após a administração intravenosa de fluorodeoxiglicose marcada com flúor-18, em jejum e seguindo uma dieta rica em gordura e pobre em carboidratos por 24 horas.

A ressonância magnética cardíaca (RMC) demonstrou presença de edema e realce tardio multifocal de padrão meso subepicárdico não isquêmico, com predomínio nas regiões ântero-septal, ínfero-septal, inferior e médio-basal inferolateral do ventrículo esquerdo, compatível com miocardiopatia inflamatória (Figura 3), e a tomografia por emissão de pósitrons - tomografia computadorizada (PET-CT), solicitada para avaliar possível sarcoidose no coração transplantado, demonstrou discreta captação heterogênea e multifocal do radiofármaco nas paredes ínfero-septal basal, inferior e ínfero-lateral do ventrículo esquerdo (SUVmáx: 2,4) (Figura 4). Além da captação cardíaca, o exame também demonstrou linfonodo intraparotídeo direito (SUVmáx: 2,9), inespecífico, provavelmente de natureza inflamatória ou reativa.

Devido à possibilidade de reativação da SC no coração transplantado, a decisão foi iniciar o tratamento visando o diagnóstico mais provável, com base na história cardíaca prévia do paciente, achados de ressonância magnética cardíaca e PET-CT, com evidência de manifestação extracardíaca de sarcoidose, e a exclusão de alternativas menos prováveis. A dose de corticosteroide foi aumentada para 0,5 mg/kg por dia durante 3 meses. Não foram feitas alterações na terapia imunossupressora basal, pois o paciente já estava recebendo doses-alvo de micofenolato mofetil e ciclosporina, com esta última mantida em um nível sérico adequado. O ecocardiograma transtorácico de controle após a terapia inicial com corticosteroides mostrou melhora da FEVE, de 45% para 57%.

## Discussão

Em indivíduos com sarcoidose sistêmica, o pulmão é o órgão mais frequentemente envolvido, sendo afetado em até 90% dos casos. Embora 20% dos pacientes com sarcoidose sistêmica encaminhados para exames de imagem apresentem envolvimento cardíaco, a doença clinicamente manifesta é encontrada em apenas 5%.^1^ Outros locais de envolvimento incluem o sistema linfático, manifestações cutâneas, sistema ocular, fígado e baço, sistema neurológico e musculoesquelético e glândulas salivares, com aumento de volume, particularmente das glândulas parótidas.^1^

Embora o TC seja uma terapia definitiva para a SC em estágio terminal com insuficiência cardíaca refratária ou arritmias com risco de vida, a recorrência da SC no coração transplantado apresenta desafios diagnósticos e de manejo,^2,3^ como ilustrado neste caso. A recorrência da SC pós-transplante é um fenômeno raro, variando de 5% a 18% na literatura. Apesar da baixa prevalência, o potencial de recorrência reforça a necessidade de vigilância a longo prazo e diferenciação de outras causas de disfunção do enxerto, como a rejeição celular.^2,4^

Após o TC, os dados sugerem que os pacientes com SC apresentam taxas de sobrevivência semelhantes ou até mesmo melhoradas em comparação com outras etiologias de insuficiência cardíaca, desde que os regimes imunossupressores sejam cuidadosamente conduzidos.^4^ Entretanto, pequenos estudos e relatos de casos indicam que a recorrência de sarcoidose no aloenxerto é possível e associada a ajustes de imunossupressão ou descontinuação de corticosteroides.^4^

A recorrência da SC ou rejeição celular pós-transplante pode apresentar características sobrepostas, incluindo o surgimento ou agravamento dos sintomas de insuficiência cardíaca, arritmias ou redução da FEVE. Ao contrário da rejeição celular, que normalmente se manifesta de forma aguda, a recorrência da SC costuma se apresentar de forma mais insidiosa, com achados de imagem precedendo os sintomas clínicos.^3^

A diferenciação entre recorrência de SC e rejeição celular requer uma combinação de dados clínicos, de imagem e histopatológicos.^3^

1. Tomografia por emissão de pósitrons - tomografia computadorizada (PET-CT): A PET-CT surgiu como uma ferramenta altamente sensível para detectar inflamação miocárdica, incluindo SC recorrente. A tomografia por emissão de pósitrons com 18-fluoro-desoxiglicose (18F-FDG PET) pode identificar a atividade metabólica na inflamação granulomatosa, mesmo antes de ocorrerem alterações estruturais.^5^ Em casos de sarcoidose recorrente, a PET-CT frequentemente revela áreas irregulares de aumento da captação de FDG, correspondendo a inflamação granulomatosa. Embora a PET-CT não seja específica para sarcoidose, sua capacidade de detectar inflamação a torna um complemento valioso para o monitoramento da recorrência da doença, particularmente na ausência de outro envolvimento sistêmico.^5^
  Como exemplo de manifestação extracardíaca, o envolvimento das glândulas salivares, particularmente das glândulas parótidas, é uma manifestação reconhecida, mas relativamente incomum, da sarcoidose. Ocorre em aproximadamente 6 a 30% dos pacientes com sarcoidose sistêmica, e a 18F-FDG PET pode detectar aumento da captação nas glândulas afetadas, embora tais achados sejam frequentemente inespecíficos e possam mimetizar infecção, neoplasia ou outras condições inflamatórias,^6^ como descrito no presente caso.
2. RMC: O realce tardio com gadolínio (RTG) no miocárdio é indicativo de fibrose ou inflamação ativa. Achados de RMC, como RTG subepicárdico ou de parede média, são comumente associados à sarcoidose. No entanto, esses achados não são patognomônicos, e a confirmação por biópsia é frequentemente necessária.^2^
3. Biópsia Endomiocárdica (BEM): O padrão ouro para o diagnóstico tanto de rejeição celular quanto de recorrência de SC. A biópsia desempenha um papel crucial na exclusão de rejeição celular, que se caracteriza por infiltração linfocitária e dano aos miócitos, em vez de inflamação granulomatosa. Em casos de recorrência de SC, granulomas podem ser identificados, embora com menor sensibilidade nas biópsias do ventrículo direito.^2^
  A sensibilidade da BEM para o diagnóstico de SC é reconhecidamente baixa, variando de <20% a 35% em diferentes séries. Isso reflete a natureza focal da lesão histopatológica típica, ou seja, o granuloma.^7^
  A ausência de granulomas na BEM realizada para avaliar episódios de rejeição aguda não pode descartar definitivamente a possibilidade de um resultado falso-negativo em nosso caso. Diferenciar a recorrência de SC da rejeição celular é importante para orientar o tratamento, visto que as estratégias de manejo diferem, especialmente no que diz respeito à intensidade da imunossupressão (Tabela 1).

**Tabela 1:** Diferenças entre recorrência de SC e rejeição celular

Recurso	Recorrência de SC	Rejeição Celular
Fisiopatologia	Granulomas não caseosos por desregulação imunológica	Inflamação do aloenxerto imunomediada
Imagem	Captação irregular de FDG (PET CT), RTG (RMC)	Disfunção global ou regional na ecocardiografia
Histopatologia	Inflamação granulomatosa	Infiltração linfocítica e necrose dos miócitos
Apresentação Clínica	Início insidioso, arritmias, redução da FEVE	Início agudo, insuficiência cardíaca, sintomas sistêmicos
Resposta à terapia	Escalonamento de corticoides, ajuste imunossupressor	Terapia imunossupressora aumentada

SC: sarcoidose cardíaca; PET CT: tomografia por emissão de pósitrons - tomografia computadorizada; RTG: realce tardio com gadolínio; RMC: ressonância magnética cardíaca; FEVE: fração de ejeção do ventrículo esquerdo.

Os regimes imunossupressores pós-TC geralmente incluem inibidores de calcineurina (tacrolimus ou ciclosporina), micofenolato de mofetila e corticosteroides. Os corticosteroides são essenciais na prevenção da recorrência da SC devido aos seus efeitos anti-inflamatórios e são iniciados em doses de 30 a 40 mg/dia de equivalente de prednisona, pois não há benefício demonstrado com doses iniciais mais altas.^3^ Relatórios sugerem que a descontinuação ou redução gradual dos corticosteroides pode desencadear a recorrência da SC, conforme observado em estudos anteriores nos quais a terapia de manutenção em baixas doses pareceu reduzir o risco de recorrência.^8^ A rejeição celular, por outro lado, frequentemente requer intensificação da terapia imunossupressora ou corticosteroides em dose pulsada para controlar a inflamação e prevenir a perda do enxerto.^8^

## Conclusão

Este caso destaca os desafios diagnósticos e terapêuticos do manejo da redução da FEVE de início recente em um receptor de TC, onde a diferenciação entre recorrência de SC e rejeição celular é crucial. A terapia imunossupressora, particularmente corticosteroides, desempenha um papel fundamental no manejo de ambas as condições, sendo necessárias abordagens personalizadas com base na etiologia subjacente.

## Disponibilidade de Dados

Os conteúdos subjacentes ao texto da pesquisa estão contidos no manuscrito.
