# Supplementary material for: Reativação da Sarcoidose Cardíaca em um Coração Transplantado: Um Caso Incomum de Nova Disfunção do Enxerto
Source: Arq Bras Cardiol. 2025 Nov 27;122(10):e20250311. [Article in Portuguese] doi: 10.36660/abc.20250311 (PMC12674836; doi:10.36660/abc.20250311)
Supplement: Translation

# Translation: Cardiac Sarcoidosis Reactivation in a Transplanted Heart: An Unusual Case of New Graft Dysfunction

**DOI:** 10.36660/abc.20250311i

**Authors:** Manuela Cristina Ribeiro Dias Barroso, Heleutério da Conceição Nicolau Madogolele, Danielle Louvet Guazzelli, Luis Fernando Bernal da Costa Seguro, Fabiana Goulart Marcondes-Braga, Sandrigo Mangini, Monica Samuel Ávila, Gabriel Barros Aulicino, Iascara Wozniak Campos, Samuel Padovani Steffen, Fabio Antonio Gaiotto, Fernando Bacal

**Affiliations:** 1 São Paulo SP Brazil Instituto do Coração do Hospital das Clínicas da Faculdade de Medicina da Universidade de São Paulo, São Paulo, SP – Brazil

**Keywords:** Sarcoidosis, Heart Transplantation, Primary Graft Dysfunction

## Introduction

Cardiac sarcoidosis (CS) is an uncommon yet potentially life-threatening manifestation of systemic sarcoidosis. Recurrence of CS following heart transplantation (HTx) is an exceedingly rare event and presents a significant diagnostic challenge.

## Case Report

A 50-year-old female patient admitted to our service due to a new graft dysfunction, with left ventricular ejection fraction (LVEF) reduction on a routine transthoracic echocardiogram (from 64% to 45%), was asymptomatic.

CS was diagnosed at age 39, first manifested with high-degree atrioventricular block with pacemaker implantation at age 37. The patient developed advanced heart failure with reduced ejection fraction, with multiple hospitalizations, and remained symptomatic despite optimal medical therapy. At the age of 39, bicaval orthotopic HTx was performed.

The patient experienced a smooth and uneventful early recovery following HTx, with consistently negative surveillance endomyocardial biopsy (EMB) results and gradual titration of cyclosporine. Long-term immunosuppressive therapy included cyclosporine 50mg twice a day, mycophenolate mofetil 500mg twice a day, and prednisone 5mg a day.

Given the new reduction in LVEF in a transplanted heart, the hypotheses of graft rejection, occult infection, cardiac allograft vasculopathy (CAV), and CS reactivation were considered.

Initial blood tests revealed C-reactive protein was <0.4mg/L [NR: 1-3mg/L], B-type natriuretic peptide of 380pg/mL [NR: <100pg/mL], ultrasensitive troponin I of 17ng/L [NR < 16ng/L], and cyclosporine serum level of 210ng/mL. Electrocardiogram in sinus rhythm, previous right bundle branch block, and normal cardiothoracic index in the chest x-ray, with metal wires from previous sternotomy.

Further investigation for a potential occult infectious focus, including imaging studies, blood cultures, and urine culture, yielded negative results. An EMB was then performed, which revealed 1R cellular rejection and no evidence of humoral rejection (Figure 1), and panel-reactive antibody showed no donor-specific antibodies. At the cardiac catheterization, there was no evidence of CAV (Figure 2).

**Figure 1:** Photomicrographies of the last endomyocardial biopsy showing (a) Absence of inflammatory infiltrate or fibrosis. (b) Minimal focus of myocardial injury (black arrows) and mild fibrosis (F). The degree of acute cellular rejection was 1R. The fragments were sectioned into several levels, and there was no evidence of granulomas. Haematoxylin-Eosin staining, objective magnifications respectively 10X and 20X.

**Figure 2:** Invasive coronary angiography. (a) Right anterior oblique view showing the right coronary artery with no significant obstructive lesions. (b) Left anterior oblique caudal view showing the left coronary artery, with the left anterior descending and circumflex arteries free of critical stenosis.

Cardiac magnetic resonance imaging (MRI) showed presence of edema and multifocal late enhancement of nonischemic meso subepicardial pattern, with predominance in the anteroseptal, inferoseptal, inferior and mid-basal inferolateral left ventricle, compatible with inflammatory cardiomyopathy (Figure 3), and positron emission tomography - computed tomography (PET-CT), requested to evaluate possible sarcoidosis in the transplanted heart, demonstrated discrete heterogeneous and multifocal uptake of the radiopharmaceutical in the basal inferoseptal, inferior and inferolateral walls of the left ventricle (SUVmax: 2.4) (Figure 4). In addition to cardiac uptake, the scan also demonstrated a right intraparotid lymph node (SUVmax: 2.9), nonspecific, most likely inflammatory or reactive in nature.

**Figure 3:** Cardiac MRI showing edema and multifocal late enhancement of nonischemic meso subepicardial pattern, with predominance in the inferoseptal, inferior, and mid-basal inferolateral left ventricle, compatible with inflammatory cardiomyopathy (red arrows).

**Figure 4:** ^18^F-Fluorodeoxyglucose positron emission tomography/computed tomography demonstrating (red arrows) mild, heterogeneous, and multifocal radiotracer uptake in the basal inferoseptal, inferior, and inferolateral segments of the left ventricular myocardium (maximum standardized uptake value: 2.4), findings suggestive of an ongoing active inflammatory process. The images were acquired using a positron emission tomography scanner coupled with computed tomography (64-slice CT scanner), one hour after intravenous administration of fluorine-18-labeled fluorodeoxyglucose, under fasting conditions and following a 24-hour high-fat, low-carbohydrate diet.

Due to the possibility of reactivation of CS in the transplanted heart, the decision was to initiate treatment targeting the most likely diagnosis, based on the patient's prior cardiac history, cardiac MRI, and PET-CT findings, with evidence of extracardiac manifestation of sarcoidosis, and the exclusion of less probable alternatives. Corticosteroid dose was increased to 0.5 mg/kg per day for 3 months. No changes were made to the baseline immunosuppressive therapy, as the patient was already receiving target doses of mycophenolate mofetil and cyclosporine, with the latter maintained at an appropriate serum level. Control transthoracic echocardiogram after initial corticosteroid therapy showed LVEF improvement, from 45% to 57%.

## Discussion

In individuals with systemic sarcoidosis, the lung is the most frequently involved organ, affected in up to 90% of cases. Although 20% of patients with systemic sarcoidosis referred for imaging have cardiac involvement, clinically manifest disease is encountered in only 5%.^1^ Other sites of involvement include the lymphatic system, cutaneous manifestations, ocular system, liver and spleen, neurological and musculoskeletal system, and salivary glands, with enlargement, particularly of the parotid glands.^1^

While HTx is a definitive therapy for end-stage CS with refractory heart failure or life-threatening arrhythmias, recurrence of CS in the transplanted heart poses diagnostic and management challenges,^2,3^ as illustrated in this case. Post-transplant CS recurrence is a rare phenomenon, varying from 5% to 18% in the literature. Despite the low prevalence, the potential for recurrence underscores the need for long-term surveillance and differentiation from other causes of graft dysfunction, such as cellular rejection.^2,4^

Following HTx, data suggest that CS patients have similar or even improved survival rates compared to other heart failure etiologies, provided immunosuppressive regimens are carefully managed.^4^ However, small studies and case reports indicate that recurrence of sarcoidosis in the allograft is possible and associated with immunosuppression adjustments or discontinuation of corticosteroids.^4^

CS recurrence or cellular rejection post-transplant can present with overlapping features, including new-onset or worsening heart failure symptoms, arrhythmias, or reduced LVEF. Unlike cellular rejection, which typically manifests acutely, CS recurrence often presents more insidiously, with imaging findings preceding clinical symptoms.^3^

Differentiating between CS recurrence and cellular rejection requires a combination of clinical, imaging, and histopathological data.^3^

1. Positron Emission Tomography - computed tomography (PET-CT): PET-CT has emerged as a highly sensitive tool for detecting myocardial inflammation, including recurrent CS. 18-Fluoro-deoxyglucose Positron Emission Tomography (18F-FDG PET) can identify metabolic activity in granulomatous inflammation, even before structural changes occur.^5^ In cases of recurrent CS, PET-CT imaging often reveals patchy areas of increased FDG uptake corresponding to granulomatous inflammation. While PET-CT is not specific for sarcoidosis, its ability to detect inflammation makes it a valuable adjunct for monitoring disease recurrence, particularly in the absence of other systemic involvement.^5^
  As an example of extracardiac manifestation, salivary gland involvement, particularly of the parotid glands, is a recognized but relatively uncommon manifestation of sarcoidosis. It occurs in approximately 6–30% of patients with systemic sarcoidosis, and 18F-FDG PET can detect increased uptake in affected glands, although such findings are often nonspecific and may mimic infection, neoplasm, or other inflammatory conditions,^6^ as described in the present case.
2. Cardiac MRI: Late gadolinium enhancement (LGE) in the myocardium is indicative of fibrosis or active inflammation. Cardiac MRI findings, such as subepicardial or mid-wall LGE, are commonly associated with sarcoidosis. However, these findings are not pathognomonic, and biopsy confirmation is often necessary.^2^
3. Endomyocardial Biopsy (EMB): The gold standard for diagnosing both cellular rejection and CS recurrence. Biopsy plays a critical role in ruling out cellular rejection, which is characterized by lymphocytic infiltration and myocyte damage rather than granulomatous inflammation. In cases of CS recurrence, granulomas may be identified, albeit with lower sensitivity in right ventricular biopsies.^2^
  The sensitivity of EMB for the diagnosis of CS is known to be low, varying from <20% to 35% in different series. This reflects the focal nature of the typical histopathological lesion, that is, the granuloma.^7^
  Absence of granulomas in the EMB performed to evaluate acute rejection episodes cannot definitively rule out the possibility of a false-negative result in our case. Differentiating CS recurrence from cellular rejection is important for guiding treatment, as the management strategies differ, especially when it comes to intensity of immunosuppression (Table 1).

**Table 1:** Differences between CS recurrence and cellular rejection

Feature	CS Recurrence	Cellular Rejection
Pathophysiology	Non-caseating granulomas from immune dysregulation	Immune-mediated allograft inflammation
Imaging	Patchy FDG uptake (PET CT), LGE (cardiac MRI)	Global or regional dysfunction on echocardiography
Histopathology	Granulomatous inflammation	Lymphocytic infiltration and myocyte necrosis
Clinical Presentation	Insidious onset, arrhythmias, reduced LVEF	Acute onset, heart failure, systemic symptoms
Response to Therapy	Corticosteroid escalation, immunosuppressive adjustment	Augmented immunosuppressive therapy

CS: cardiac sarcoidosis; PET CT: positron emission tomography - computed tomography; LGE: late gadolinium enhancement; MRI: magnetic resonance imaging; LVEF: left ventricular ejection fraction.

Immunosuppressive regimens post-HTx typically include calcineurin inhibitors (tacrolimus or cyclosporine), mycophenolate mofetil, and corticosteroids. Corticosteroids are critical in preventing CS recurrence due to their anti-inflammatory effects, and are initiated at doses of 30 to 40 mg/d of prednisone equivalent because there is no demonstrated benefit with higher starting doses.^3^ Reports suggest that discontinuation or tapering of corticosteroids may trigger CS recurrence, as seen in prior studies where low-dose maintenance therapy appeared to reduce recurrence risk.^8^ Cellular rejection, in contrast, often requires intensification of immunosuppressive therapy or pulse-dose corticosteroids to control inflammation and prevent graft loss.^8^

## Conclusion

This case highlights the diagnostic and therapeutic challenges of managing new-onset LVEF reduction in a heart transplant recipient, where differentiation between CS recurrence and cellular rejection is crucial. Immunosuppressive therapy, particularly corticosteroids, plays a pivotal role in managing both conditions, with tailored approaches needed based on the underlying etiology.

## Data Availability

The underlying content of the research text is contained within the manuscript.
